# Supplementary material for: Transsynaptic mapping of Drosophila mushroom body output neurons
Source: eLife. 2021 Feb 11;10:e63379. doi: 10.7554/eLife.63379 (PMC7877909; doi:10.7554/eLife.63379)
Supplement: Supplementary file 1. [file elife-63379-supp1.docx]

| **Figure** | **Experiment** | **n** | **Statistical Test** | **Result** | **Post-hoc** | **Result** |
| --- | --- | --- | --- | --- | --- | --- |
| 1A | MB011B-split-GAL4/*trans-*Tango | Female: n=2  Male: n=3 | N/A | N/A | N/A | N/A |
| 1B | MB002B-split-GAL4/*trans-*Tango | Female: n=7  Male: n=2 | N/A | N/A | N/A | N/A |
| 1C | MB399B-split-GAL4/*trans-*Tango | Female: n=1  Male: n=9 | N/A | N/A | N/A | N/A |
| 1D | MB310C-split-GAL4/*trans-*Tango | Female: n=2  Male: n=3 | N/A | N/A | N/A | N/A |
| 1E | MB434B-split-GAL4/*trans-*Tango | Female: n=5  Male: n=4 | N/A | N/A | N/A | N/A |
| 1F | MB298B-split-GAL4/*trans-*Tango | Female: n=8  Male: n=1 | N/A | N/A | N/A | N/A |
| 1G | MB110C-split-GAL4/*trans-*Tango | Female: n=3  Male: n=3 | N/A | N/A | N/A | N/A |
| 1H | MB057B-split-GAL4/*trans-*Tango | Female: n=8  Male: n=2 | N/A | N/A | N/A | N/A |
| 1I | MB077B-split-GAL4/*trans-*Tango | Female: n=3  Male: n=5 | N/A | N/A | N/A | N/A |
| 1J | MB018B-split-GAL4/*trans-*Tango | Female: n=3  Male: n=8 | N/A | N/A | N/A | N/A |
| 1K | MB026B-split-GAL4/*trans-*Tango | Female: n=3  Male: n=4 | N/A | N/A | N/A | N/A |
| 1L | MB080C-split-GAL4/*trans-*Tango | Female: n=3  Male: n=4 | N/A | N/A | N/A | N/A |
| 1M | MB082C-split-GAL4/*trans-*Tango | Female: n=2  Male: n=2 | N/A | N/A | N/A | N/A |
| 1N | MB542B-split-GAL4/*trans-*Tango | Female: n=3  Male: n=3 | N/A | N/A | N/A | N/A |
| 1O | MB050B-split-GAL4/*trans-*Tango | Female: n=4  Male: n=3 | N/A | N/A | N/A | N/A |
| 1P | MB051C-split-GAL4/*trans-*Tango | Female: n=3  Male: n=3 | N/A | N/A | N/A | N/A |
| 1Q | MB549C-split-GAL4/*trans-*Tango | Female: n=4  Male: n=3 | N/A | N/A | N/A | N/A |
| 1R | MB027B-split-GAL4/*trans-*Tango | Female: n=5  Male: n=4 | N/A | N/A | N/A | N/A |
| S1.1A | MB074C-split-GAL4/*trans-*Tango | Female: n=2  Male: n=7 | N/A | N/A | N/A | N/A |
| S1.1B | MB210B-split-GAL4/*trans-*Tango | Female: n=3  Male: n=3 | N/A | N/A | N/A | N/A |
| S1.1C | MB433B-split-GAL4/*trans-*Tango | Female: n=3  Male: n=3 | N/A | N/A | N/A | N/A |
| S1.1D | MB083C-split-GAL4/*trans-*Tango | Female: n=7  Male: n=3 | N/A | N/A | N/A | N/A |
| S1.1E | MB051C-split-GAL4/*trans-*Tango | Female: n=3  Male: n=3 | N/A | N/A | N/A | N/A |
| S1.1F | MB077C-split-GAL4/*trans-*Tango | Female: n=3  Male: n=2 | N/A | N/A | N/A | N/A |
| 3A | MB011B-split-GAL4/*trans-*Tango | Male hemibrains: n=4 | N/A | N/A | N/A | N/A |
| 3B | MB002B-split-GAL4/*trans-*Tango | Male hemibrains: n=4 | N/A | N/A | N/A | N/A |
| 3C | MB074C-split-GAL4/*trans-*Tango | Male hemibrains: n=4 | N/A | N/A | N/A | N/A |
| 3D | MB083C-split-GAL4/*trans-*Tango | Male hemibrains: n=10 | N/A | N/A | N/A | N/A |
| 3E | MB077C-split-GAL4/*trans-*Tango | Male hemibrains: n=8 | N/A | N/A | N/A | N/A |
| S3.1A | MB310C-split-GAL4/*trans-*Tango | Male hemibrains: n=4 | N/A | N/A | N/A | N/A |
| S3.1B | MB433B-split-GAL4/*trans-*Tango | Male hemibrains: n=4 | N/A | N/A | N/A | N/A |
| S3.1C | MB298B-split-GAL4/*trans-*Tango | Male hemibrains: n=4 | N/A | N/A | N/A | N/A |
| S3.1D | MB057B-split-GAL4/*trans-*Tango | Male hemibrains: n=4 | N/A | N/A | N/A | N/A |
| S3.1E | MB027B-split-GAL4/*trans-*Tango | Male hemibrains: n=4 | N/A | N/A | N/A | N/A |
| S3.1F | MB080C-split-GAL4/*trans-*Tango | Male hemibrains: n=4 | N/A | N/A | N/A | N/A |
| S3.1G | MB542B-split-GAL4/*trans-*Tango | Male hemibrains: n=4 | N/A | N/A | N/A | N/A |
| 7A | R47H090-LexA; MB077C/*UAS-CD2,*  *QUAS-mtdTomato*; LexAop-GFP | Male: n=6 | N/A | N/A | N/A | N/A |
| 7B | VT055139-LexA; MB077C-split-GAL4/ *UAS-CD2,*  *QUAS-mtdTomato*; LexAop-GFP | Male: n=4 | N/A | N/A | N/A | N/A |
| 7C | MB077C>47H09  MB077C>VT055139 | n=5  n=6 | N/A | N/A | N/A | N/A |
| 7D | R47H09-LexA; MB083C-split-GAL4/ *UAS-CD2,*  *QUAS-mtdTomato*; LexAop-GFP | Male: n=2 | N/A | N/A | N/A | N/A |
| 7E | VT055139-LexA; MB083C-split-GAL4/ *UAS-CD2,*  *QUAS-mtdTomato*; LexAop-GFP | Male: n=2 | N/A | N/A | N/A | N/A |
| 7G | +/SS32219  shits/SS32219  shits/+ | n=8 (10 flies per n)  n=8 (10 flies per n)  n=8 (10 flies per n) | One-way ANOVA | F(2,21)=39.28, p<0.0001 | N/A | N/A |
| 7H | Pathlength | +/SS32219 (n=10)  shits/SS32219 (n=10)  shits/+ (n=10) | One-way ANOVA | F(2,29)=33.39, p<0.0001 | Tukey  Tukey  Tukey | +/SS32219 vs shi^ts^/SS32219 p= 0.0000  shi^ts^/SS32219 vs  shi^ts^/+ p=0.0000  +/SS32219 vs +/shi^ts^ p=0.1932 |
| 7H | Angular velocity | +/SS32219 (n=10)  shits/SS32219 (n=10)  shits/+ (n=10) | One-way ANOVA | F(2,29)=51.87, p<0.0001 | Tukey | +/SS32219 vs shi^ts^/SS32219 p= 0.0000  shi^ts^/SS32219 vs  shi^ts^/+ p=0.0000  +/SS32219 vs +/shi^ts^ p=0.0680 |
| 7H | Velocity | +/SS32219 (n=10)  shits/SS32219 (n=10)  shits/+ (n=10) | One-way ANOVA | F(2,29)=30.97, p<0.0001 | Tukey | +/SS32219 vs shi^ts^/SS32219 p= 0.0000  shi^ts^/SS32219 vs  shi^ts^/+ p=0.0000  +/SS32219 vs +/shi^ts^ p=0.3280 |
| S7.1 | MB077C>VT055139 | n=2 | N/A | N/A | N/A | N/A |
| S.7.2A | +/SS32230  shits/ SS32230  shits/+ | n=8 (10 flies per n)  n=8 (10 flies per n)  n=8 (10 flies per n) | One-way ANOVA | F(2,21)=13.94 p<0.0001 | N/A | N/A |
| S7.2B | Pathlength | +/SS32230 (n=10)  shi^ts^/SS32230 (n=10)  shi^ts^/+ (n=10) | One-way ANOVA | F(2,28)=14.76, p<0.0001 | Tukey | +/ SS32230 vs shi^ts^/ SS32230 p= 0.0000  shi^ts^/ SS32230 vs  shi^ts^/+ p=0.0000  +/ SS32230 vs +/shi^ts^ p=0.1932 |
| S7.2B | Angular velocity | +/SS32230 (n=10)  shi^ts^/SS32230 (n=10)  shi^ts^/+ (n=10) | One-way ANOVA | F(2,28)=28.16, p<0.0001 | Tukey | +/SS32230 vs shi^ts^/ SS32230 p= 0.0000  shi^ts^/SS32230 vs  shi^ts^/+ p=0.0000  +/SS32230 vs +/shi^ts^ p=0.0680 |
| S7.2B | Velocity | +/SS32230 (n=10)  shi^ts^/SS32230 (n=10)  shi^ts^/+ (n=10) | One-way ANOVA | F(2,28)=13.96, p<0.0001 | Tukey | +/SS32230 vs shi^ts^/SS32230 p= 0.0000  shi^ts^/SS32230 vs  shi^ts^/+ p=0.0000  +/SS32230 vs +/shi^ts^ p=0.3280 |
| S7.3A | +/SS32219  shi^ts^/SS32219  shit^s^/+ | n=8 (10 flies per n)  n=8 (10 flies per n)  n=8 (10 flies per n) | One-way ANOVA | F(2,21)=4.617, p=0.02 | Tukey | +/SS32219 vs shi^ts^/SS32219 p= 0.08  shi^ts^/SS32219 vs  shi^ts^/+ p=0.82  +/SS32219 vs +/shi^ts^ p=0.02 |
| S7.3B | +/SS32230  shi^ts^/SS32230  shi^ts^/+ | n=8 (10 flies per n)  n=8 (10 flies per n)  n=8 (10 flies per n) | One-way ANOVA | F(2,21)=6.195, p=0.0078 | Tukey | +/SS32230 vs shi^ts^/SS32230 p= 0.97  shi^ts^/SS32230 vs  shi^ts^/+ p=0.013  +/SS32230 vs +/shi^ts^ p=0.02 |

Supplementary Table 1. Statistical Analysis Summary
